# 4189 The Changing Health and Social Circumstances of Women Leaving Jails: A Three-year Longitudinal Study

**DOI:** 10.1017/cts.2020.431

**Published:** 2020-07-29

**Authors:** Stephanie Assimonye, Megha Ramaswamy, Jason Glenn, Sharla Smith

**Affiliations:** 1 University of Kansas Frontiers; 2 University of Kansas Medical Center

## Abstract

OBJECTIVES/GOALS: To characterize the various social and health trajectories of women released from jail, and how these trajectories influence women’s risky sexual and drug behaviors. To identify areas in which prevention programs and community interventions can be implemented to improve social and health outcomes. METHODS/STUDY POPULATION: The present study analyzes data collected as part of the sexual health empowerment (SHE Project) health literacy intervention. Participants were recruited from three county jails in the greater Kansas City area. At baseline, participants completed a survey that assessed participants’ sociodemographic characteristics and social histories prior to incarceration. Women were recruited between 2014-2016 and followed up annually after program completion to complete follow-up surveys to assess long-term health and social circumstances. The present study is a secondary analysis of baseline and follow-up data. Final analyses will include survey data from 126 women. RESULTS/ANTICIPATED RESULTS: In this study, we use Hobfoll’s Conservation of Resources (COR) Theory to conceptualize the impacts of stress on the social and health behaviors of justice-involved women in the years following release from jail. We hypothesize that “loss spirals”, a term coined by Stevan Hobfoll, creates psychological stress that drive justice-involved women to assume behaviors that will generate more resources and help to cope with the stress. We expect to find that women struggle to maintain ties to stable housing, employment, and support, which we believe to be central to “loss spirals.” Additionally, we expect to find that these “loss spirals” are associated with sexual and drug health risks. DISCUSSION/SIGNIFICANCE OF IMPACT: This study aims to define a succinct longitudinal timeline assessing biopsychosocial outcomes of women released from jail in order to improve prevention and intervention techniques for the improvement in social and health circumstances of women leaving jail and their reduction in recidivism.

